# The fungicide azoxystrobin promotes freshwater cyanobacterial dominance through altering competition

**DOI:** 10.1186/s40168-019-0744-0

**Published:** 2019-09-04

**Authors:** Tao Lu, Qi Zhang, Michel Lavoie, Youchao Zhu, Yizhi Ye, Jun Yang, Hans W. Paerl, Haifeng Qian, Yong-Guan Zhu

**Affiliations:** 1 College of Environment, Zhejiang University of Technology, Hangzhou, 310032 People’s Republic of China; 2 Quebec-Ocean and Takuvik Joint International Research Unit, Université Laval, G1VOA6, Québec, Canada; 3 Key Laboratory of Urban Environment and Health, Institute of Urban Environment, Chinese Academy of Sciences, Xiamen, 361021 People’s Republic of China; 4 Institute of Marine Sciences, University of North Carolina at Chapel Hill, Morehead City, NC 28557 USA; 5 College of Environment, Hohai University, Nanjing, 210098 People’s Republic of China; 6 State Key Laboratory of Desert and Oasis Ecology, Xinjiang Institute of Ecology and Geography, Chinese Academy of Sciences, Urumqi, 830011 People’s Republic of China; 7 State Key Lab of Urban and Regional Ecology, Research Center for Ecoenvironmental Sciences, Chinese Academy of Sciences, Beijing, 100085 People’s Republic of China

**Keywords:** Fungicide, Eutrophication, Algal bloom, Meta-transcriptomics, Indirect effects

## Abstract

**Background:**

Sharp increases in food production worldwide are attributable to agricultural intensification aided by heavy use of agrochemicals. This massive use of pesticides and fertilizers in combination with global climate change has led to collateral damage in freshwater systems, notably an increase in the frequency of harmful cyanobacterial blooms (HCBs). The precise mechanisms and magnitude of effects that pesticides exert on HCBs formation and proliferation have received little research attention and are poorly constrained.

**Results:**

We found that azoxystrobin (AZ), a common strobilurin fungicide, can favor cyanobacterial growth through growth inhibition of eukaryotic competitors (Chlorophyta) and possibly by inhibiting cyanobacterial parasites (fungi) as well as pathogenic bacteria and viruses. Meta-transcriptomic analyses identified AZ-responsive genes and biochemical pathways in eukaryotic plankton and bacteria, potentially explaining the microbial effects of AZ.

**Conclusions:**

Our study provides novel mechanistic insights into the intertwined effects of a fungicide and eutrophication on microbial planktonic communities and cyanobacterial blooms in a eutrophic freshwater ecosystem. This knowledge may prove useful in mitigating cyanobacteria blooms resulting from agricultural intensification.

**Electronic supplementary material:**

The online version of this article (10.1186/s40168-019-0744-0) contains supplementary material, which is available to authorized users.

## Background

In the past century, agricultural intensification supported by the use of agrochemicals increased human food supply to keep pace with a rapidly increasing population on Earth. However, this massive use of pesticides and inorganic fertilizers in combination with global climate change has led to extensive collateral damage in aquatic ecosystems, most notably an increase in the frequency of harmful cyanobacterial blooms (HCBs) [1, 2]. HCBs lead to bottom water oxygen depletion and cyanotoxin accumulation (microcystins, anatoxins, etc.) with negative effects on diverse ecosystems, including habitat loss and economic costs (loss of fisheries, recreational use, and tourism) as well as human health risks [3–5]. Cyanobacterial dynamics on a short-time scale (weeks) are related to a range of abiotic factors [6], such as nutrient availability, light, temperatures and precipitation patterns [7], and biotic factors, including allelopathic interactions between cyanobacteria and other microbial species [8, 9]. Several persistent organic pollutants, including pesticides, are also increasingly recognized as a potential contributing factor for cyanobacterial blooms [10, 11], but the underlying mechanisms at the molecular level remain largely unknown.

Fungicide residues in aquatic environments, which usually co-exists with human-induced eutrophication, can inhibit the growth of fungi, eukaryotic algae, and some zooplankton species [12–14]. Cyanobacterial populations are controlled by many biotic factors, including viral and bacterial attack, fungal parasitism, zooplankton grazing and allelopathic interactions with various microbes [15]. Here, we investigate the possibility that fungicide contamination could inhibit the growth of cyanobacterial competitors and potentially contribute to cyanobacterial dominance and HCBs.

Azoxystrobin (AZ), a globally distributed fungicide ($1.165 billion in global sales reported for 2016) (http://cn.agropages.com/, accessed on October 19, 2017), is a broad-spectrum strobilurin fungicide that protects food crops against many pathogenic fungi. The extensive use of AZ has led to contamination of nearby freshwater ecosystems at concentrations reaching 0.01–29.70 μg L^−1^ in streams, ponds, groundwater, and lakes in Denmark, Germany, France, Brazil, and the USA [14, 16, 17]. AZ can have toxic effects on a number of aquatic organisms [14, 18, 19]. Eukaryotic algae and fungi are particularly sensitive to AZ, compared to cyanobacteria, potentially affecting microbial community structure and enhancing cyanobacterial growth [20].

Here, we study the effects of AZ on microbial community composition in microcosms containing water from a large eutrophic freshwater Chinese lake. We show that AZ favors cyanobacterial bloom species, and we unravel the previously unrecognized physiological and molecular mechanisms underlying community change using meta-transcriptomic analyses. Our study suggests that AZ application can paradoxically worsen HCBs through modulating microbial interactions.

## Materials and methods

### Aquatic microcosm set up and AZ exposure

A series of 2-L water samples was collected from 0.5-m depth in Lake Taihu (March, 2017), which is located in the Yangtze Basin, bordering Shanghai, Jiangsu, and Zhejiang provinces in the southeastern part of China (30°55′40″–31°32′58″ N; 119°52′32″–120°36′10″ E). The water samples were filtered through a 0.22 μm polycarbonate membrane (Jinjing^TM^, Shanghai, China). Filtered material was resuspended in a small volume of sterile nutrient-enriched BG-11 medium (initial pH = 7.1, chemical composition in Additional file 1: Table S1) to prepare the microbial community stock cultures (MTC). The medium containing macronutrients was autoclaved at 121 °C for 25 min. The trace metal solution was filter-sterilized beforehand and then mixed with macronutrients. The MTC solution was then inoculated in the microcosms, consisting of 2 L of modified BG-11 medium contained in a beaker. The optical density of all replicated microcosms at 680 nm, a proxy for algal biomass, was adjusted to 0.01, i.e., ca. 0.07 mg Chl-a L^−1^. After MTC inoculation, AZ was added in the microcosms at initial concentrations between 0.5 and 25 mg L^−1^. The microcosms were placed in an artificial greenhouse at 25 ± 0.5 °C under cool-white fluorescent light (46 μmol photons m^−2^ s^−1^) with a 12-h:12-h light: dark cycle. Because other pollutants, including pesticides, exist in natural lake water, the experiments involving transfer of the plankton community from lake water to a pesticide-free artificial medium were meant to isolate and asses the effect of one stressor (AZ addition) on the communities under a controlled condition.

### Microalgae monoculture and AZ growth inhibition

Axenic strains of the green algae *Chlorella pyrenoidosa* (FACHB-9) and *Monoraphidium* sp. (FACHB-1853), as well as of the cyanobacterium *Microcystis aeruginosa* (FACHB-905) and *Synechococcus* sp. (FACHB-805), obtained from the Institute of Hydrobiology at the Chinese Academy of Sciences (Wuhan, China), were cultivated at the same condition as those used for the microcosms. The tested species were maintained in exponential growth in batch cultures in 250-mL Erlenmeyer glass flasks containing 150 mL of modified BG-11 medium. All cultures were manually agitated three times a day. The cell density of the culture was measured every 24 h, using a spectrophotometer at an optical density of 680 nm (OD_680_). Standard curves that expressed cell density as a function of OD_680_ for each alga were generated using standardized algal culture and a hemocytometer [20, 21]. The initial cell density was set at 600,000 and 200,000 cells/mL for *C. pyrenoidosa* and *M. aeruginosa*, respectively. AZ were added at different initial concentrations between 0.5 and 5 mg L^−1^. Algal growth inhibition over time at a given AZ concentration was calculated as follows: % inhibition = 100 × [(*A*_control_ − *A*_exposed_)/*A*_control_], where *A*_exposed_ and *A*_control_ are the cell densities at times 2 to 7 days in the AZ-treated and control cultures, respectively.

### Analysis of photosynthetic pigments

After 72 h, the microcosms’ chlorophyll a (Chl-a) contents were measured as described by Inskeep and Bloom [22] in microcosms exposed to 0–25 mg L^−1^ AZ (*n* = 4). In parallel, phycocyanin was extracted in a sodium phosphate buffer and measured by spectrophotometry according to the methodology detailed in Silveira et al. [23] (*n* = 4).

### Measurement of dissolved azoxystrobin

Residual AZ concentration was determined in the dissolved phase of the batch cultures or microcosms (after 0, 7, and 15 days) by solid-phase extraction-HPLC (high-performance liquid chromatography) using the following methodology (*n* = 4). Five-milliliter microcosm samples were successively passed through a 0.45-μm aperture pinhole filter and a solid-phase extraction apparatus with WondaSep C18 column. The column was eluted with 1 mL of acetonitrile three times. The eluates were collected in a 10-mL centrifuge tube and diluted with acetonitrile. AZ was finally quantified by HPLC using a 250-nm UV detection wavelength at 25 °C with Acetonitrile:Water (68:32) as the mobile phase.

### Co-cultivation of *Synechococcus* sp. and *Monoraphidium* sp.

*Synechococcus* sp. and *Monoraphidium* sp. were grown together in batch cultures for 7 days. Co-cultivation was carried out in both the modified BG-11 medium and filtered eutrophic lake water. The chemical composition of inorganic nutrients in modified BG-11 medium was the same as that in microcosms. Regarding the co-cultures in lake water, the lake water was autoclaved and filtered through a 0.22-μm polycarbonate membrane (Jinjing^TM^, Shanghai, China) and N and P were added using NH_4_NO_3_ and KH_2_PO_4_ autoclaved stock solution (final N and P concentrations in lake water were adjusted to 6 mg L^−1^ and 0.3 mg L^−1^, respectively). Cell number was calculated using a hemocytometer (*n* = 20).

### Meta-transcriptomic sample preparation and sequencing

Three control microcosms (Con1, Con2, and Con3) as well as three other microcosms with 2.5 mg L^−1^ AZ (initial concentration) (AZ1, AZ2, AZ3) were harvested after 7 days of culture for the meta-transcriptomic work. Microbial biomass in the six aquatic microcosms was centrifuged at 6500 g for 5 minutes at 4 °C and then the supernatant was processed by gentle filtration on a 0.2-μm filter. The microbial community collected on the filter as well as the centrifugal precipitation were used for RNA extraction. Total RNA was purified using TRIzol Reagent (Invitrogen, Thermo fisher, USA) and the RNeasy Mini Kit (Qiagen, Germany). RNA was quantified and characterized using an Agilent 2100 Bioanalyzer (Agilent Technologies, Palo Alto, CA, USA), and a NanoDrop (Thermo Fisher Scientific Inc.). Gel electrophoresis (1% agarose formaldehyde) was used as an RNA integrity test. One microgram total RNA with a RIN (RNA integrity number) value above seven was used for library preparation. The ribosomal depleted mRNA was then fragmented, reverse-transcribed and the sequences were processed and analyzed by GENEWIZ (Suzhou, China). Transcriptome sequencing (RNA-seq) was performed on an Illumina HiSeq 4000 platform. The raw data was quality trimmed, assembled, and annotated as detailed in Additional file 1: Extended Materials and Methods. Detailed statistics of clean data and sample transcripts from six microcosms are shown in Additional file 2: Dataset 1.

### Data analysis

We used FPKM (expected number of fragments per kilobase of transcript sequence per millions base pairs sequenced) to calculate relative transcript level of genes. All experiments were repeated three times independently. Data are presented as means ± standard errors. Significant differences among treatments were tested with one-way ANOVAs (StatView 5.0). The ANOVA assumptions of normality and homogeneity of variance of residuals were validated with the Kolmogorov-Smirnov one-sample test and Levene’s test, respectively (StatView 5.0). Differences were considered statistically significant when *p* < 0.05.

## Results and discussion

### Toxicity of AZ on green algae and cyanobacteria

**Fig. 1:**
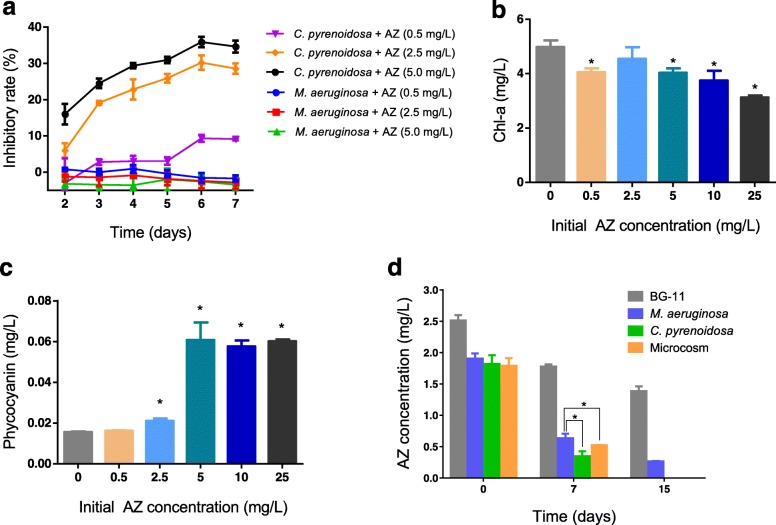
Influence of azoxystrobin (AZ) on microalgae. **a** Growth inhibition of *Chlorella pyrenoidosa* and *Microcystis aeruginosa* grown in batch cultures over 2–7 days in the presence of 0.5, 2.5, or 5 mg L^−1^ initial AZ concentration. Chl-a (**b**) and phycocyanin (**c**) concentration in microcosms after a 3-day AZ exposure. **d** Dissolved AZ concentrations (nominal initial concentration = 2.5 mg L^−1^) in BG-11 medium, batch cultures of *C. pyrenoidosa* and *M. aeruginosa*, and in the microcosm over time. Asterisks (*) denote significant differences (*p* < 0.05) compared to the first column in panels **b** and **c**

Model cyanobacterium *M. aeruginosa* as well as a common green alga *C. pyrenoidosa* were used to investigate the toxicity of AZ on green algae and cyanobacteria. Growth of *M. aeruginosa* was not suppressed by the range of AZ concentrations during the 7-day treatment (Fig. 1a), while *C. pyrenoidosa* growth was inhibited by approximately 9.2–30% at the three tested AZ concentrations after 7 days (Fig. 1a). Growth of *C. pyrenoidosa* in the presence of a low AZ concentration (5–10 μg L^−1^) and at low initial algal cell density (about 20,000 cells/mL, close to the algal density in nature), which are conditions representative of naturally contaminated environments [14, 16, 17], was inhibited significantly by 20~30% (Additional file 1: Figure S2) (*p* < 0.05), while *M. aeruginosa* growth remained unaffected at the same tested AZ concentrations. Previous laboratory studies also showed that AZ inhibitory toxic effects varied dramatically between green algae and cyanobacteria; for instance, the toxicity of dissolved AZ on the growth of the chlorophyte *Pseudokirchneriella subcapitata* was nearly 500-fold higher than that reported in the cyanobacterium *Anabaena flos-aquae* [24, 25].

Microcosms were used to study the effects of AZ to the plankton community. Microorganisms were separated from natural lake water by filtration and transferred to an artificial medium, after which a range of AZ concentrations was added to the microcosms. Concentrations of Chl-a and phycocyanin were measured in the microcosms after AZ treatment. The Chl-a concentration, which estimates total phytoplankton biomass [26], reached 4 mg L^−1^ in the control microcosms after 3 days of culture in the medium and a phytoplankton bloom occurred (Fig. 1b). Exposure to AZ concentration higher or equal than the lowest concentration tested (0.5 mg L^−1^) for 3 days decreased Chl-a concentration, indicating that AZ can have toxic effects on the phytoplankton populations comprising the green algae (Fig. 1b). Chl-a concentration decreased by 8.7% to 37.3% over the range of AZ concentrations tested in microcosms. In contrast, production of another pigment, phycocyanin, which was used as a proxy for cyanobacterial biomass [26], increased after the 3-day exposure at concentrations of AZ higher than or equal to 2.5 mg L^−1^ (Fig. 1c), suggesting that AZ favored cyanobacterial growth in the microcosm.

The rate of azoxystrobin decomposition in the aquatic environment is stimulated by light and AZ has an aqueous photolysis DT_50_ (time to 50% dissipation) (pH 7) between 8.7 and 13.9 days [14]. Interestingly, dissolved AZ concentrations decreased differently over time in response to green algae and cyanobacteria; decreasing more rapidly in *C. pyrenoidosa* than in *M. aeruginosa* cultures (Fig. 1d). This indicated that the eukaryotic alga *C. pyrenoidosa* took up more dissolved AZ than *M. aeruginosa*, even though they were inoculated at the same optical density. Depletion of the dissolved AZ concentration in the microcosms was also more rapid than in the *M. aeruginosa* cultures (Fig. 1d). AZ depletion in the microcosms could be explained by preferential AZ uptake/adsorption in green alga (the major component in microcosms) as well as higher AZ adsorption on other aquatic microorganisms except cyanobacteria. The above results suggest that *C. pyrenoidosa* was much more sensitive to AZ than *M. aeruginosa*, probably due in part to a higher AZ consumption (uptake/adsorption) in *C. pyrenoidosa*.

### Transcripts proportions variation after AZ exposure in the whole plankton community

Meta-transcriptomic sequencing was carried out to investigate the changes in transcription of the whole plankton community after AZ exposure. A summary of the meta-transcriptomic sequencing results is provided in Additional file 1: Extended Results. Taxonomic proportions of transcripts in two groups at different taxonomic levels are shown in Additional file 2: Dataset 2, which were represented by the relative abundance of taxonomically annotated transcripts (RAT). The RAT value does not represent microbial biomass, but rather the changes in transcriptional activity among species, which represents the active metabolic states and functions of the microbial community.

**Fig. 2.**
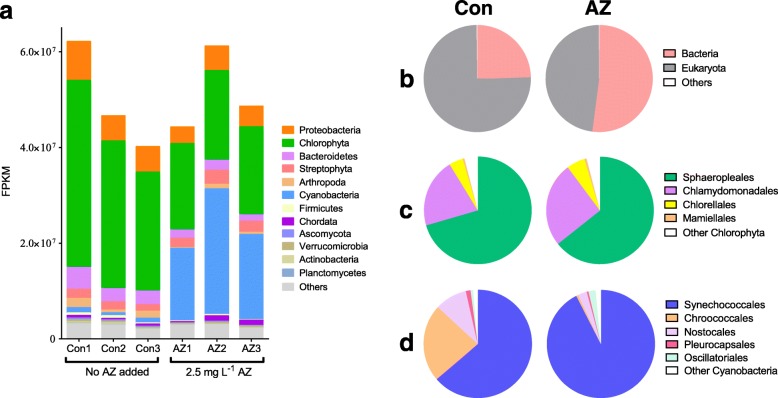
Taxonomic proportions of transcripts in the control or azoxystrobin (AZ) group. **a** Expected number of fragments per kilobase of transcript sequence per millions base pairs sequenced (FPKM) of the six microcosms calculated from meta-transcriptomic analysis indicating major taxonomic groups (phylum level) in the microcosms with no added azoxystrobin (Control group) and microcosms with 2.5 mg L^−1^ (AZ group) for 7 days. **b** Taxonomic proportions of transcripts in different kingdoms in the control or AZ group. Taxonomic proportions of transcripts of different orders within the division of *Chlorophyta* (**c**) and *Cyanobacteria* (**d**) in the control or AZ group

The relative abundance of RAT in control microcosms after 7 days of culture was mainly controlled by *Monoraphidium* sp. (a *Chlorophyta* genus) (Fig. 2). However, the relative abundance of RAT of *Chlorophyta* decreased from 63.6% in the control to 35.8% in AZ-treated microcosms (Fig. 2a, Additional file 1: Table S2) even though the taxonomic transcript counts of major classes inside the *Chlorophyta* phylum was not affected much by AZ treatment (Fig. 2c). The relative abundance of RAT of other eukaryotic algal species among the *Phaeophyceae* and *Eustigmatophyceae* also decreased significantly in the microcosms treated with AZ (*p* < 0.05), while the abundance of *Bacillariophyta* increased by ~ 6-fold (Additional file 1: Table S2). Meanwhile, the RAT of *Cyanobacteria* (mainly composed of *Synechococcales*) dramatically increased by more than 20-fold, i.e., from 1.7% in the control group to 38.3% in the AZ group. The sequence abundance ratio of eukaryota/prokaryota decreased from 3.1 to 0.9 (Fig. 2b) in the AZ-treated microcosms mainly due to the drastic increase of *Cyanobacteria* and the decrease of *Chlorophyta*, respectively. The rise in *Cyanobacteria* in AZ-treated microcosms was coupled to a higher proportion of *Synechococcales* and a lower proportion of *Chroococcales* (Fig. 2d), whereas AZ did not affect the relative abundance of orders within the Chlorophyta, the main eukaryotic division (Fig. 2c). Cyanobacteria within the order *Synechococcale* can form toxic blooms, potentially affecting eukaryotic algal competitors [27–29].

### AZ inhibits both *Monoraphidium* sp. and *Synechococcus* sp. in monocultures

**Fig. 3:**
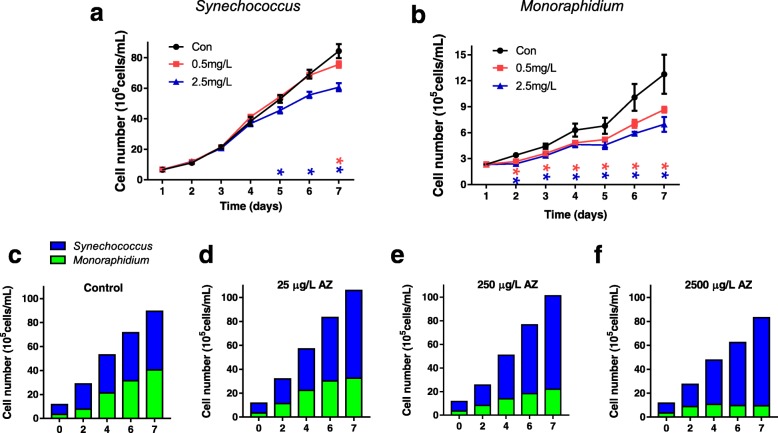
Monoculture and co-cultivating of *Synechococcus* sp. and *Monoraphidium* sp. in response to AZ. Algal cell number of *Synechococcus* sp. (**a**) and *Monoraphidium* sp. (**b**) grown in batch cultures for 1 to 7 days with no added azoxystrobin (AZ) or with 0.5 - 2.5 mg L^−1^ AZ. Algal cell number of *Synechococcus* sp. and *Monoraphidium* sp. co-cultured in lake water for 7 days with the initial AZ concentration 0 (**c**), 25 μg L^−1^ (**d**), 250 μg L^−1^ (**e**), and 2.5 mg L^−1^ (**f**). N and P concentrations in lake water were adjusted to 6 mg L^−1^ and 0.3 mg L^−1^, respectively. Cell number was calculated by a hemocytometer (*n* = 20)

As the RAT of *Monoraphidium* sp. was halved and the RAT of *Synechococcus* sp. sharply increased after AZ treatment, AZ toxicity bioassays in laboratory batch cultures of *Synechococcus* sp. and *Monoraphidium* sp. were carried out to strengthen our hypothesis that AZ addition in the microcosms could preferentially benefit a cyanobacterium (*Synechococcus* sp.) over a green algal competitor (*Monoraphidium* sp.). Interestingly, the growth of monocultures of *Synechococcus* sp. in BG-11 medium as inhibited by ~ 28% after 7 days (Fig. 3a) (*p* < 0.05) to an initial AZ concentration of 2.5 mg L^−1^, although it was not significantly influenced after 4 days of exposure (*p* = 0.21), indicating that *Synechococcus* sp. growth could be inhibited by long-term exposure to high AZ concentrations. In contrast, the growth of *Monoraphidium* in laboratory monoalgal cultures was always inhibited throughout the entire cultivation process; with a cell yield inhibited by ~ 45% after 7 days of exposure to 2.5 mg L^−1^ AZ (initial concentration) (Fig. 3b). The above results indicate that *Monoraphidium* sp. is more sensitive to AZ than *Synechococcus* sp. Due to the remarkable growth advantage of *Synechococcus* sp. in the AZ-treated microcosms (Fig. 2d), it appears that AZ plays an indirect role in benefiting *Synechococcus* sp.

### AZ inhibits *Monoraphidium* sp. and benefits *Synechococcus* sp. in co-cultures

When co-cultivating the two algae species, it was found that the addition of AZ benefits *Synechococcus* sp. over *Monoraphidium* sp. in both filtered eutrophic lake water (Fig. 3c–f) and modified BG-11 medium (Additional file 1: Figure S3), which was in accordance with the RAT results of meta-transcriptomic analysis. After a 7-day culture in eutrophic lake water, the cell number ratio (*Synechococcus*/*Monoraphidium*) was enhanced by the AZ treatment, from 1.2 in the control to 2.3, 3.6, or 7.7, for the treatment with 25 μg L^−1^, 250 μg L^−1^, or 2.5 mg L^−1^ AZ, respectively (Fig. 3c–f). The results of prokaryotic and eukaryotic algal growth in modified BG-11 medium (Additional file 1: Figure S3) were similar to that in eutrophic lake water, where the *Synechococcus* sp. gain dominance in the AZ treatment. There exists a balance between green algae and cyanobacteria in natural water, driven by several factors including allelopathic interactions and positive feedback [8, 30–33]. The presence of AZ disturbed this balance, probably through altering the metabolism of green algae [20], and stimulated *Synechococcus* sp. growth relative to that of *Monoraphidium* sp. in the co-cultures (Fig. 3c–f). After AZ treatment, the cell number of *Synechococcus* sp. increased to ~ 1.5-fold in co-culture (Fig. 3c–f) while the RAT of *Synechococcus* sp. increased more than 20-fold in microcosm compared to control. Since RAT cannot be easily compared to cell density, it is difficult to quantitatively compare the laboratory and microcosm experiments. However, both experiments suggest that AZ favors cyanobacterial growth through altering competition with green algae.

### AZ changes the transcriptional activity of fungi, viruses, bacteria, and zooplankton

**Fig. 4:**
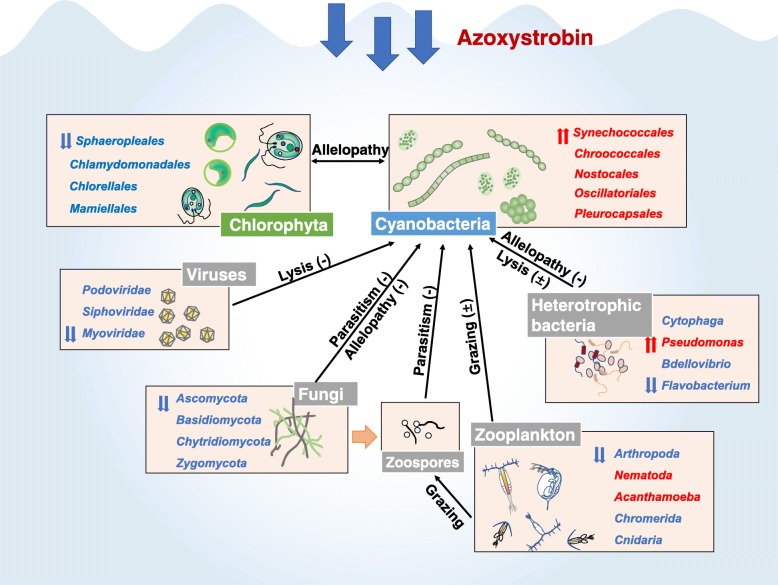
Schematic overview of the main biotic factors modulating cyanobacteria relative abundance after AZ treatment for 7 days. Biotic factors mainly include Chlorophyta, viruses, fungi, zooplankton and heterotrophic bacteria. Red or blue font indicates that the relative abundance of the organisms increased or decreased, respectively, in response to the AZ treatment relative to that of the control. Plus sign indicates potentiated effect; minus sign indicates weakened effect. Double-arrow indicates the organism that has the biggest change (value of absolute change) in this kind of factor. The changes of these natural relationships between these species and cyanobacteria are not necessarily the direct cause of the increase of cyanobacteria. They may also be the result of the cyanobacterial bloom

The RAT of fungi, including *Zygomycota*, *Basidiomycota*, *Chytridiomycota*, and *Ascomycota*, all decreased significantly (*p* < 0.05) after exposure to AZ compared to the control (Additional file 1: Table S2) due to the fungicidal action of AZ. It is worth noting that the RAT of the phylum *Chlorobi*, which includes photosynthetic bacteria that do not produce oxygen and prefer anaerobic environments, also increased significantly (~ 8-fold) (*p* < 0.05) in the AZ-treated microcosms. Although total dissolved oxygen concentration (DO) remained super-saturated in the AZ-treated microcosms compared to the control (Additional file 1: Figure S4), anoxic microzones surrounding microbial (perhaps cyanobacterial) aggregates may be well developed in the AZ-treated microcosms [34], which would be in line with the increase in the RAT of *Chlorobi*. The RAT of zooplankton in most phylum, such as Arthropoda, Nematoda, and Cnidaria, decreased under AZ exposure relative to the control (Additional file 1: Table S2), while the reverse was true for some genera like *Acanthamoeba* (Table 1). In general, the RAT of some zooplankton (such as *Daphnia*, from *Arthropoda*), fungi (belonging to *Chytridiomycota*), heterotrophic bacteria (*Cytophaga* and *Bdellovibrio*), and viruses (*Podoviridae*, *Siphoviridae*, and *Myoviridae*) significantly decreased (*p* < 0.05) in AZ-treated microcosms compared to that in the control (Table 1, Fig. 4). The above planktonic organisms can graze, parasitize or lyse cyanobacteria and play roles in controlling their abundance [15, 35–37] and hence might explain partly cyanobacteria dominance in the presence of AZ. Another possibility for the decrease in relative abundance of zooplankton is that these changes may reflect the decrease of edible chlorophytes and increase of inedible cyanobacteria, for chlorophytes have the higher sensitivity to AZ than cyanobacteria.

**Table 1:** Variations of biotic factors potentially affecting cyanobacteria growth in the microcosms exposed to azoxystrobin for 7 days (AZ) as inferred from fold change in relative abundance of taxonomic transcripts of selected organisms in the control group (Con) or the AZ-treated microcosms (AZ).

	Organisms	Relative abundance		Fold change (AZ/Con)
		Con (%)	AZ (%)	
Graze	*Daphnia*	2.60	0.915	0.35
	*Acanthamoeba*	0.0286	0.0512	1.82
Bacterial lysis	*Alcaligenes*	5.05E− 05	1.49E− 04	2.95
	*Flavobacterium*	0.413	0.205	0.50
	*Cytophaga*	0.00633	0.00190	0.30
	*Pseudomonas*	0.203	0.412	2.03
	*Bdellovibrio*	0.105	0.0105	0.10
	*Bacillus*	0.0105	0.00963	0.92
Viral lysis	*Podoviridae*	1.55E− 04	1.18E− 05	0.08
	*Siphoviridae*	6.95E− 04	3.56E− 04	0.51
	*Myoviridae*	0.00380	0.00157	0.41
Allelopathy	*Chlorophyceae*	58.7	32.9	0.56
	*Cylindrospermopsis*	0.00131	0.0351	26.9
Fungal parasitism	*Chytridiomycota*	0.0695	0.0375	0.54

It is also known that fungal zoospores play critical roles in linking inedible cyanobacteria and zooplankton. *Arthropoda* (cladocerans or copepods) are unable to synthesize de novo sterols and have to find them from their food. For instance, chytrid zoospores are an essential food complement for *Daphnia* when grazing on filamentous cyanobacteria [38, 39]. Since the fungicide AZ strongly inhibited fungi and therefore probably reduced fungal zoospores (Table 1), it is possible that *Daphnia* grazing on cyanobacteria decreases, which would in turn contribute to decreased *Daphnia* growth (Table 1) and increase cyanobacteria growth, thereby protecting cyanobacteria from being grazed. Neither the zooplankton community nor the viral and parasites of cyanobacteria were likely to control the cyanobacterial bloom. However, they can add stability to the aquatic system by modifying the food web structure [40] and can affect the microbial community as well as potentially contribute to the cyanobacterial bloom.

### Metabolic pathways in eukaryota and bacteria in response to AZ treatment

**Fig. 5.**
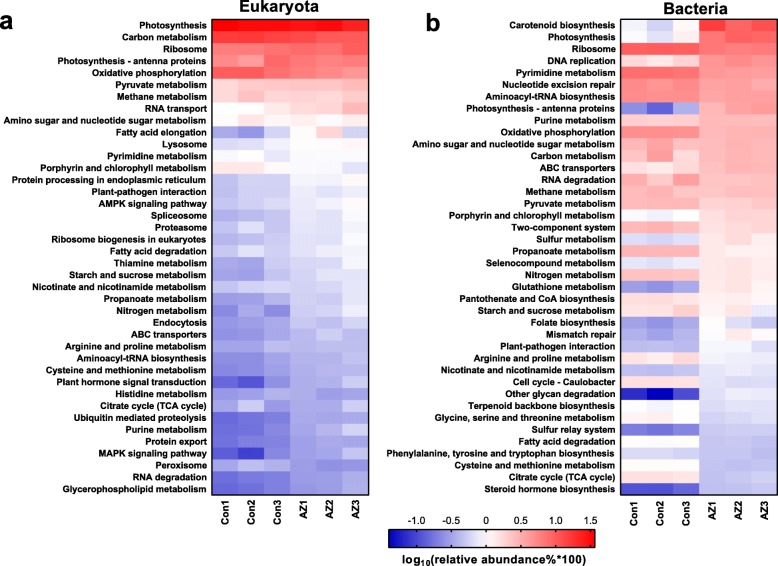
Functional variations between the control and azoxystrobin (AZ) group at KEGG level 3. Relative abundance (% expressed on a log10 basis) of the 40 most important functional categories (KEGG level 3) in eukaryote (**a**) and bacteria (**b**) of each of the six microcosms based on meta-transcriptomic data. The three control microcosms are denoted Con1, Con2, and Con3, and the three microcosms exposed to 2.5 mg L^−1^ (initial concentrations) azoxystrobin (AZ) are denoted AZ1, AZ2, and AZ3

Since overall transcriptomic annotation information is limited in the genus *Chlorella* and other Chlorophytes [41], meta-transcriptomic analyses of the entire microbial community were helpful to further understand the interactions between planktonic organisms and cyanobacteria. A summary of the functional annotation is provided in Additional file 1: Extended Results, and the detailed functional variation is provided in Additional file 2: Dataset 3, 4. We distinguished sequences of eukaryota and bacteria and dissected them at KEGG (Kyoto Encyclopedia of Genes and Genomes) level 3. Figure 5 shows the 40 most important pathways based on relative abundance of transcripts (belonging to four metabolic systems mentioned in the previous section) in eukaryota and bacteria.

Even though the composing proportion of eukaryota was not heavily affected by AZ (Additional file 1: Figure S5a), the KEGG level 3 pathways were often appreciably affected (Additional file 2: Dataset 4). As shown in Fig. 5a, the most highly over-expressed pathways due to the presence of AZ in eukaryota were plant hormone signal transduction, MAPK signaling pathway, nitrogen metabolism, ubiquitin-mediated proteolysis, and glycerophospholipid metabolism, and the most under-expressed pathways were porphyrin and chlorophyll metabolism, oxidative phosphorylation, and peroxisome, indicating that AZ modulates functional gene expression in eukaryota.

In response to the cyanobacterial bloom, the bacterial community structure and the relative abundance of metabolic pathways changed dramatically (Fig. 5b and Additional file 1: Figure S5b). As shown in Fig. 5b, pathways closely related to cyanobacteria were significantly over-expressed (*p* < 0.05), including carotenoid biosynthesis (+ 1326% compared with control), photosynthesis-antenna proteins (+ 1305%) and photosynthesis (+ 758%). We also noted that many pathways with a low relative abundance (< 0.05) related to antibiosis, vitamin metabolism, and polysaccharide synthesis were differentially expressed in response to AZ (Additional file 2: Dataset 4). These functions are discussed below.

1. Modulation of oxidative phosphorylation and photosynthesis by AZ. The relative expression level of genes related to oxidative phosphorylation in both eukaryota and prokaryota decreased dramatically in the AZ-treated microcosms, and the degree of inhibition in the two kingdoms was equivalent, i.e., ~ 42% compared to control. Genes (*atpA*, *atpB*, *atpD*, and *atpF*) coding for the enzyme F-ATPase, in the mitochondrial and chloroplastic membranes, were significantly under-expressed in both eukaryota and bacteria (decreased by around 40~80%) (*p* < 0.05). This decrease in F-ATPase transcription could be explained by the toxic action of AZ, which inhibits electron transfer complex between cytochrome b and cytochrome c1, decreasing mitochondrial respiration and ATP production [42, 43]. Regarding photosynthetic metabolic pathways, the modulation of photosystem transcripts after the AZ treatment was different between eukaryota and bacteria (Additional file 1: Figure S6). In bacteria, genes involved in phycobilisome (e.g., *apcD*, *apcE*, *cpeC*, *cpeZ*) were strongly over-expressed (by around 4–32-fold), while in eukaryota, the transcription of genes coding for proteins of the light-harvesting complex (e.g., *LHCB*2 and *LHCA*1) remained mostly unchanged (Additional file 2: Dataset 5). This is in line with the rise in cyanobacteria in the AZ-treated microcosms. It is worth noting, however, that bacteria may have AZ detoxification mechanisms to decrease AZ toxicity by a strong increase (by around 10–24-fold) of NAD(P)H-quinone oxidoreductase genes (*ndhD*, *ndhF*, *ndhH*), which encode an enzyme protecting the cells against environmental stress [44, 45] that may occur in the presence of AZ. In addition, prokaryotes may detoxify reactive oxygen species through an increased carotenoid biosynthesis (+ 1326%) and glutathione metabolism (+ 344%) and improve cellular reparation (genes involved in mismatch repair, + 211%) [46–48].
2. Polysaccharide synthesis and degradation. Transcription of several genes related to polysaccharide biosynthesis in eukaryotes increased (Additional file 1: Table S3), for instance, genes in the pathways of lipopolysaccharide biosynthesis (+353%). A polysaccharide-associated system (ABC transporter, +162%) was also over-expressed in both AZ and control group, which has been shown to export polysaccharides outside of the cells. Enhanced polysaccharide synthesis in phytoplankton has been reported in stressful environments [49, 50]. In contrast, in bacteria, 4 pathways (N-Glycan biosynthesis, various types of N-glycan biosynthesis, lipopolysaccharide biosynthesis, and peptidoglycan biosynthesis) related to glycan synthesis were under-expressed (by around 63~77%) significantly (*p* < 0.05), but the pathway “other glycan degradation” was over-expressed by ~ 10-fold in the AZ-treated microcosms compared to the control. Assuming that the generalized predicted decrease in polysaccharide synthesis in prokaryote occurred in cyanobacteria, a putative decrease in polysaccharide ballast in cyanobacteria could help maintain cyanobacteria at the lake surface and help exploit light, which is strongly attenuated with depth during the bloom. Several cyanobacteria including those of the genus *Synecocchococus* have indeed been shown to modulate buoyancy through polysaccharide synthesis [51].
3. Modulation of vitamin biosynthesis and potential interactions between bacteria and eukaryotes. In eukaryotes, all pathways related to B vitamin biosynthesis (e.g., thiamine, riboflavin, niacinamide, pantothenate, vitamin B_6_, biotin, lipoic acid, and folate) were over-expressed (by 14~243%) in the AZ-treated microcosms compared to the control (Additional file 1: Table S4). By contrast, in bacteria, biochemical pathways related to folate, nicotinamide, and thiamine biosynthesis were over-expressed (37~139%) after the AZ exposure in microcosms, while vitamin biosynthetic pathways in lower relative abundance (i.e., biotin and lipoic acid, riboflavin, B_6_) were significantly under-expressed (by 22~68%, *p* < 0.05) in the AZ-treated microcosms compared to the control (Additional file 1: Table S4). Interestingly, in bacteria, the relative abundance of two genes involved in vitamin B_12_ (cobalamin) transport (i.e., *btuB* or K16092: vitamin B_12_ transporter and *btuF* or K06858: vitamin B_12_ transport system substrate-binding protein) decreased by 88% and 57%, respectively, in the AZ-treated microcosms relative to the control (see Additional file 2: Dataset 5). Since several eukaryotic species (including fungi and many Chlorophytes) cannot synthesize cobalamin de novo and rely on mutualistic bacteria to acquire this vitamin [52, 53], our results support the hypothesis that the rise in cyanobacteria in the presence of AZ may be, at least in part, explained by a decrease in vitamin B_12_ mutualistic exchange between bacteria and eukaryotes.

### AZ changed interactions among fungi, eukaryotic algae, and cyanobacteria

Our results are consistent with allelopathic interactions between fungi or eukaryotes and prokaryotes with potential implications in cyanobacterial bloom dynamics. First, our reported decrease in the relative activity of many fungi, which produces several secondary metabolites promoting cell lysis of cyanobacteria [54], may help favor cyanobacteria. Second, two eukaryotic pathways of antibiotic biosynthesis (monobactam biosynthesis (+ 1524%) and penicillin and cephalosporin biosynthesis (+ 161%), were preferentially expressed by 1.6- to 15-fold under AZ exposure. This suggests that eukaryotic microorganisms could respond to the rise in cyanobacteria by the production of antibacterial compounds, although it is unlikely that this prevents cyanobacterial bloom outbreaks. Third, as shown in Additional file 1: Figure S7, the relative abundance of cyanobacteria in the bacterial community was 39.3% in our sampling site from Lake Taihu (deriving from 16S rRNA gene sequencing data), while the RAT of cyanobacteria in the bacteria after 7d-culture in the control and AZ-treated groups were 7.1% and 72.1%, respectively. It indicated that the large proportion of green algae (Fig. 3c) could limit the activity of cyanobacteria, even under eutrophic conditions.

### Long-term potential impact of short-term AZ contamination

As shown in Additional file 1: Figure S8, after 50-day cultivation, the algae and organic matter settled in the microcosms, rendering them clear in the control group. However, the AZ group still showed the typical characteristics of an algal bloom, appearing green and turbid. This interesting phenomenon illustrated that, although the concentration of AZ was below the detection limits after 15 days in microcosms (Fig. 1d), the high variability at the primary stage still cause lasting influence until 50 days (Additional file 1: Figure S8). The AZ residue was widely detected in water environment [14], indicating that these water bodies may have once suffered in a short-term high-concentration contamination of AZ. The dissolved AZ decreased rapidly in the microcosms (Fig. 1d), dropping below detection limit after 15 days of algal culture, which indicated that dissolved AZ was dissipated quickly in the microcosms. In natural water systems, AZ can be taken up by plankton, adsorbed onto organic surfaces and sediments, or be dissipated through biodegradation and photolysis [55]. Therefore, peak AZ concentration is expected to be much higher than the detected dissolved concentrations in lakes, streams, or groundwater from 0.01 to 29.70 μg L^−1^ [14], suggesting that fungicide concentrations close to those used in our experiments may well transiently occur which would cause long-term negative effects. For instance, the residues of fungicide Thiram® could be detected in the range of 0.27–2.52 mg L^−1^ from surface water around the applied plots [56]. Furthermore, various other fungicides that can interact with AZ exist in aquatic systems (often adsorbed to sediments) and may be released back into surface water through sediment remobilization [57–59]. It follows that high AZ concentrations in the environments (at least sporadically) might induce toxic effects on aquatic microorganisms, while paradoxically, favoring cyanobacterial growth.

Fungicide contamination always co-exists with nutrient over-enrichment in waterbodies close to agricultural regions, where cyanobacterial blooms frequently occur. We can presume that the eutrophication and AZ contaminant would simultaneously occur in a limited water area near croplands after rainfall, which would cause dramatic changes of microbial community structure and promote cyanobacterial blooms. These cyanobacteria may be transferred to other water bodies following the next rainfall. They will then change the community structure of contaminated waters and contribute to eutrophication of nearby waters. It is known that the microbial ecological network has its own balance, which could be altered by eutrophication. This study indicates that fungicides may play an important role in promoting HCBs through complex community network interactions.

## Conclusions

Agricultural intensification is unfortunately coupled to pesticide use and nutrient over-enrichment, which, in combination with global climate change, is of great concern for HCBs. In this study, we have explored a novel pathway of cyanobacterial dominance or bloom formation in which fungicide can promote cyanobacterial dominance in eutrophic microcosms through preferential constraints on Chlorophyta growth and inhibition of fungi and some kinds of viruses, zooplankton, and heterotrophic bacteria, thus indirectly protecting cyanobacteria from being lysed, parasitized or grazed. This allows cyanobacteria to take advantage under fungicide contamination. Meta-transcriptomic analyses suggest that fungicide may increase prokaryotic polysaccharide consumption, decrease prokaryotic vitamin biosynthesis and exchange to negatively affect eukaryotic community. The identification of AZ-responsive genes through meta-transcriptomic analyses provides new insights into the interactive effects of a fungicide on microbial communities and cyanobacteria in eutrophic freshwater environment. Our results have important implications for understanding of cyanobacterial bloom formation as well as its mitigation. This calls for a well-managed use of fungicides as well as implementation of novel sustainable agricultural practices in order to control and mitigate HCBs.

## Additional files

Additional file 1: **Extended Materials and Methods** & **Extended Results**. **Figure S1.** Comparison of gene expressions. **Figure S2.** Growth states of algae with a low AZ concentration. **Figure S3.** Co-cultivating of *Synechococcus* and *Monoraphidium*. **Figure S4.** Dissolved oxygen concentration in microcosms. **Figure S5.** Taxonomic proportions of transcripts in eukaryotic and bacterial community. **Figure S6.** Gene expression variations in photosynthesis – antenna proteins. **Figure S7.** Bacterial community variation after 7d-culture. **Figure S8.** Long-term impact of AZ contamination. **Figure S9.** Functional variations between the control and azoxystrobin (AZ) group at KEGG level 2. **Table S1.** Chemical composition of the Modified BG11 medium. **Table S2.** Taxonomic proportions of FPKM normalized transcript counts at the phylum level in the control and AZ-treated microcosms. **Table S3.** Variation of pathways related to glycan synthesis and degradation. **Table S4.** Variation of pathways related to vitamin synthesis and consumption. (DOCX 701 kb) (DOCX 672 kb)

Additional file 2: **Dataset 1.** Detailed statistics of clean data, sample transcript and FPKM interval from six microcosms. **Dataset 2.** Taxonomic proportions in each microcosm. **Dataset 3.** Annotation and relative abundance of KOs in each microcosm. **Dataset 4**. Comparison in abundances between control and AZ groups at KEGG level 3. **Dataset 5**. Comparison in abundances between control and AZ groups at KEGG level 4. (XLSX 1138 kb)

## Data Availability

All data generated in this study are included in the article (and its supplementary information). The bacterial community composition of water samples derived from Taihu was provided in Additional file 1: Extended Materials and Methods. The raw meta-transcriptomic sequence data have been submitted to the NCBI Sequence Read Archive (SRA) database with accession numbers SAMN10362734 to SAMN10362736, and SAMN10644312 to SAMN10644314.

## Abbreviations

AZ: Azoxystrobin
Chl-a: Chlorophyll a
FPKM: Expected number of fragments per kilobase of transcript sequence per millions base pairs sequenced
HCBs: Harmful cyanobacterial blooms
HPLC: High-performance liquid chromatography
KEGG: Kyoto Encyclopedia of Genes and Genomes
MTC: Microbial community stock cultures
OD_680_: Optical density of 680 nm
RAT: Relative abundance of taxonomically annotated transcripts
RIN: RNA integrity number
