# Supplementary material for: The fungicide azoxystrobin promotes freshwater cyanobacterial dominance through altering competition
Source: Microbiome. 2019 Sep 4;7:128. doi: 10.1186/s40168-019-0744-0 (PMC6727577; doi:10.1186/s40168-019-0744-0)
Supplement: Supplementary file 1 — Extended Materials and Methods & Extended Results. Figure S1. Comparison of gene expressions. Figure S2. Growth states of algae with a low AZ concentration. Figure S3. Co-cultivating of Synechococcus and Monoraphidium. Figure S4. Dissolved oxygen concentration in microcosms. Figure S5. Taxonomic proportions of transcripts in eukaryotic and bacterial community. Figure S6. Gene expression variations in photosynthesis – antenna proteins. Figure S7. Bacterial community variation after 7d-culture. Figure S8. Long-term impact of AZ contamination. Figure S9. Functional variations between the control and azoxystrobin (AZ) group at KEGG level 2. Table S1. Chemical composition of the Modified BG11 medium. Table S2. Taxonomic proportions of FPKM normalized transcript counts at the phylum level in the control and AZ-treated microcosms. Table S3. Variation of pathways related to glycan synthesis and degradation. Table S4. Variation of pathways related to vitamin synthesis and consumption. (DOCX 701 kb) (DOCX 672 kb) [file 40168_2019_744_MOESM1_ESM.docx]

Additional File 1 for

**The Fungicide azoxystrobin promotes freshwater cyanobacterial dominance through altering competition**

Tao Lu, Qi Zhang, Michel Lavoie, Youchao Zhu, Yizhi Ye, Jun Yang, Hans W Paerl, Haifeng Qian^*^, Yong-Guan Zhu^*^

^*^Correspondence authors. Email: hfqian@zjut.edu.cn (H.F.Q.), ygzhu@iue.ac.cn (Y.G.Z.)

**This file includes:**

Extended Materials and Methods & Extended Results

Figure S1. Comparison of gene expressions.

Figure S2. Growth states of algae with a low AZ concentration.

Figure S3. Co-cultivating of *Synechococcus* and *Monoraphidium*.

Figure S4. Dissolved oxygen concentration in microcosms.

Figure S5. Taxonomic proportions of transcripts in eukaryotic and bacterial community.

Figure S6. Gene expression variations in photosynthesis – antenna proteins.

Figure S7. Bacterial community variation after 7d-culture.

Figure S8. Long-term impact of AZ contamination.

Figure S9. Functional variations between the control and azoxystrobin (AZ) group at KEGG level 2.

Table S1. Chemical composition of the Modified BG11 medium.

Table S2. Taxonomic proportions of FPKM normalized transcript counts at the phylum level in the control and AZ-treated microcosms.

Table S3. Variation of pathways related to glycan synthesis and degradation.

Table S4. Variation of pathways related to vitamin synthesis and consumption.

**Extended Materials and Methods**

***Library Preparation and Sequencing***

Total RNA of each sample was extracted using TRIzol Reagent (Invitrogen)/RNeasy Mini Kit (Qiagen). RNA was then quantified and qualified by Agilent 2100 Bioanalyzer (Agilent Technologies, Palo Alto, CA, USA), NanoDrop (Thermo Fisher Scientific Inc.) and 1% agrose gel. 1 μg total RNA with RIN (RNA Integrity Number) value above 7 was used for following library preparation. Next generation sequencing library preparations were constructed according to the manufacturer’s protocol (NEBNext® Ultra™ Directional RNA Library Prep Kit for Illumina®). The rRNA was depleted from total RNA using Ribo-Zero rRNA Removal Kit (Bacteria) (Illumina). The ribosomal depleted mRNA was then fragmented and reverse-transcribed. First strand cDNA was synthesized using ProtoScript II Reverse Transcriptase with random primers and Actinomycin D. The second-strand cDNA was synthesized using Second Strand Synthesis Enzyme Mix（include dACG-TP/dUTP）. The purified double-stranded cDNA by AxyPrep Mag PCR Clean-up (Axygen) was then treated with End Prep Enzyme Mix to repair both ends and add a dA-tailing in one reaction, followed by a T-A ligation to add adaptors to both ends. Size selection of Adaptor-ligated DNA was then performed using AxyPrep Mag PCR Clean-up (Axygen), and fragments of ~360 bp (with the approximate insert size of 300 bp) were recovered. The dUTP-marked second strand was digested with Uracil-Specific Excision Reagent (USER) enzyme (New England Biolabs). Each sample was then amplified by PCR for 11 cycles using P5 and P7 primers, with both primers carrying sequences which can anneal with flow cell to perform bridge PCR and P7 primer carrying a six-base index allowing for multiplexing. The PCR products were cleaned up using AxyPrep Mag PCR Clean-up (Axygen), validated using an Agilent 2100 Bioanalyzer (Agilent Technologies, Palo Alto, CA, USA), and quantified by Qubit 2.0 Fluorometer (Invitrogen, Carlsbad, CA, USA).

After that, libraries with different indices were multiplexed and loaded on an Illumina HiSeq instrument according to manufacturer’s instructions (Illumina, San Diego, CA, USA). Sequencing was carried out using a 2x150 paired-end (PE) configuration; image analysis and base calling were conducted by the HiSeq Control Software (HCS) + OLB + GAPipeline-1.6 (Illumina) on the HiSeq instrument.

***Data Analysis and gene annotation***

Raw shotgun sequencing reads were trimmed using cutadapt v1.9.1. Low-quality reads, N- rich reads and adapter-polluted reads were removed. Then host contamination reads were removed. The PE reads were assembled using Trinity. Trinity uses the de Bruijn graph strategy to assemble the transcriptome. Open reading frames (ORFs) were identified using TransDecoder program, with default parameters. The sequence reads from six samples were aligned to the assembled transcriptome individually, using the aligner bowtie2.The resulting files were then quantified using RSEM (v1.2.4) with default parameters to get gene-based raw hit-count data. Differential expression analysis used the DESeq Bioconductor package, a model based on the negative binomial distribution. After adjustment with the Benjamini and Hochberg’s approach for controlling the false discovery rate, a cut-off P-value of <0.05 was selected to detect differentially transcribed genes. BLASTX was used to search the protein sequences of the predicted genes with the NCBI non-redundant (Nr) protein database, CAZy database, eggNOG database and KEGG database with E–value cut off of 1e-5. GO-TermFinder was used in identifying Gene Ontology (GO) terms that annotate a list of enriched genes with a significant p-value less than 0.05. We used a hypergeometric test to find significantly differentially transcribed genesin KEGG (Kyoto Encyclopedia of Genes and Genomes) pathways.

***Initial bacterial composition of water samples***

The water samples derived from Taihu were filtered through 0.22 μm filter membrane and the DNA of filter mass were extracted using a DNA Isolation Kit (Biomiga, CA, USA) for analysis of the initial aquatic bacterial composition. The V3-V4 region of the 16S rRNA gene was amplified using primers 341F (CCTAYGGGRBGCASCAG) and 806R (GGACTACNNGGGTATCTAAT) in the Illumina HiSeq2500 platform. As shown in Figure S8, the initial bacterial community in the water samples (mean values of 9 test samples) were mainly composed of Cyanobacteria (39.3%), Proteobacteria (16.5%), Bacteroidetes (19.8%), Actinobacteria (17.5%) and Verrucomicrobia (2.6%). The raw sequencing data have been submitted to the NCBI Sequence Read Archive (SRA) database with accession numbers SRR8398903 to SRR8398908, and SRR8398923 to SRR8398925 (9 test samples in total).

**Extended Results**

***Metatranscriptomic sequencing results overview***

To investigate the global impact of AZ treatment on functions of microbiota at the molecular level, we used metatranscriptomic analysis. The total number of raw reads from the six samples (Con1, Con2, Con3, AZ1, AZ2, AZ3) were 855.7 million (M). In total, 838.3 M clean reads were generated after ﬁltering low-quality reads and trimming adaptor sequences from the raw reads. The processed clean reads were then mapped to the assembled genome. After assembly, 1.2 M unigenes were obtained and 41.1% of them (0.5 M) could be annotated using the KEGG database. The expected number of fragments per kilobase of transcript sequence per millions base pairs sequence (FPKM) was used to calculate relative transcript level of genes. Detailed statistics of clean data, sample transcript and FPKM interval from six microcosms was shown in Dataset 1. Compared with control group, RNA-Sequencing analyses identiﬁed 61354 genes whose expression changed signiﬁcantly (foldchange > 2, *p* < 0.05) after treatment with 2.5 mg L^-1^ LA for 7 d; 31461 of these genes were upregulated and 29893 were downregulated, which were visualized with an MA plot (Figure S1) based on the fold change and mean value of gene expression.

***Global*** ***functional changes in response to AZ exposure***

Analysis of the abundances of KEGG orthologs (KOs) identiﬁed the key functional features of the microbiota in microcosms. In total, 12845 different KOs were detected (Additional file 2: Dataset 3) and organized into 345 small metabolic pathways at KEGG level 3 and 43 metabolic subsystems at KEGG level 2. Comparing the transcripts, there was a signiﬁcant difference between the control and AZ treated groups in the relative abundance of observed functional assignments (*p* < 0.05). These subsystems belong to six basic metabolic systems at KEGG level 1. Figure S9 shows the effect of AZ on aquatic microbiota functions as determined with 22 subsystems (relative abundance > 0.2%) belonging to four metabolic systems (i.e., metabolism, genetic information processing, environmental information processing and cellular processes). Fourteen of the 22 subsystems were significantly over- or under-expressed (p < 0.05), but most subsystems (11) were over-expressed in response to AZ (Figure S9). For instance, the most highly over-expressed KEGG level 2 categories in the AZ-treated group relative to the control were metabolism of terpenoids and polyketides (+ 934% compared to control), metabolism of other amino acids (+ 212%), replication and repair (+ 172%), glycan biosynthesis and metabolism (+ 143%) and membrane transport (+ 133%). The most under-expressed subsystems were related to energy metabolism (- 27% compared to control) and cell motility (- 57%). Taken together, the observed contrasting transcription abundance proﬁles in the microcosms highlight the active metabolic subsystems of planktonic community in a cyanobacterial bloom and their response to AZ.


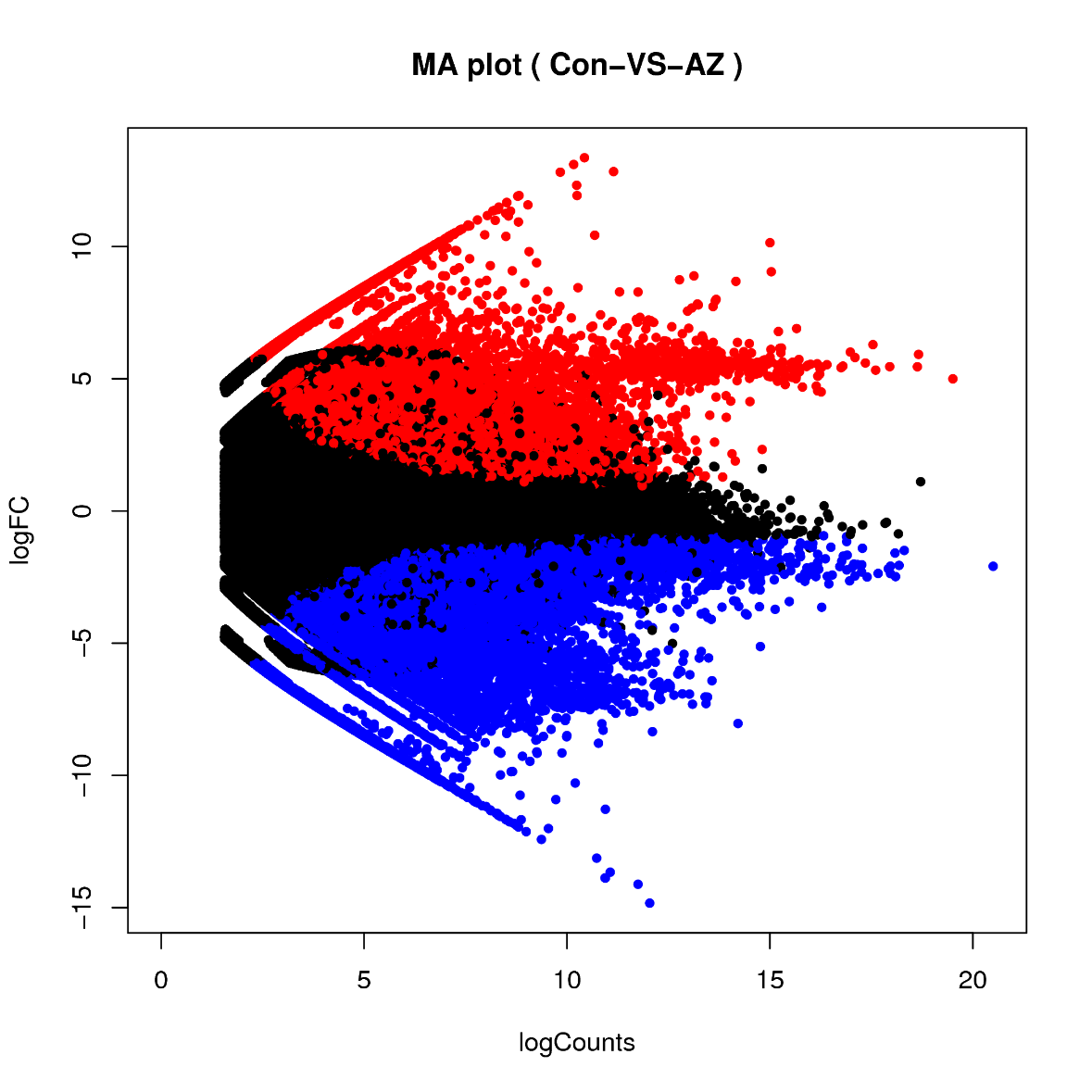


**Figure S1. Comparison of gene expressions.** MA plot (M: log ratio; A: mean average) based on the fold change in response to the 7-day azoxystrobin exposure and mean value of gene expression. The red and blue dots show the genes significantly over- and under-expressed (foldchange>2, FDR<0.05), respectively, relative to control.

**Figure S2. Growth states of algae with a low AZ concentration.** Algal cell number of *Chlorella pyrenoidosa* for 5 days of cultures (in batch) with no added AZ (Con) or with 1 - 10 μg/L initial AZ concentration. The initial cell density of the alga was approximately 20,000 cells/mL. The asterisks represent statistically signiﬁcant differences at p < 0.05 between the control and the AZ-treated group.

**Figure S3. Co-cultivating of *Synechococcus* and *Monoraphidium*.** Algal cell number of *Synechococcus* and *Monoraphidium* (co-culture) grown in batch cultures for 7 days. The initial cell number ratio (*Synechococcus*/*Monoraphidium*) was 3.3 (a) and 1.6 (b). The mixed algae were cultivated at the same condition as those described for the microcosms (see Methods section of the main text) with no added azoxystrobin (AZ) or with 2.5 mg/L AZ. Cell number was caculated by a hemocytometer (n=20).


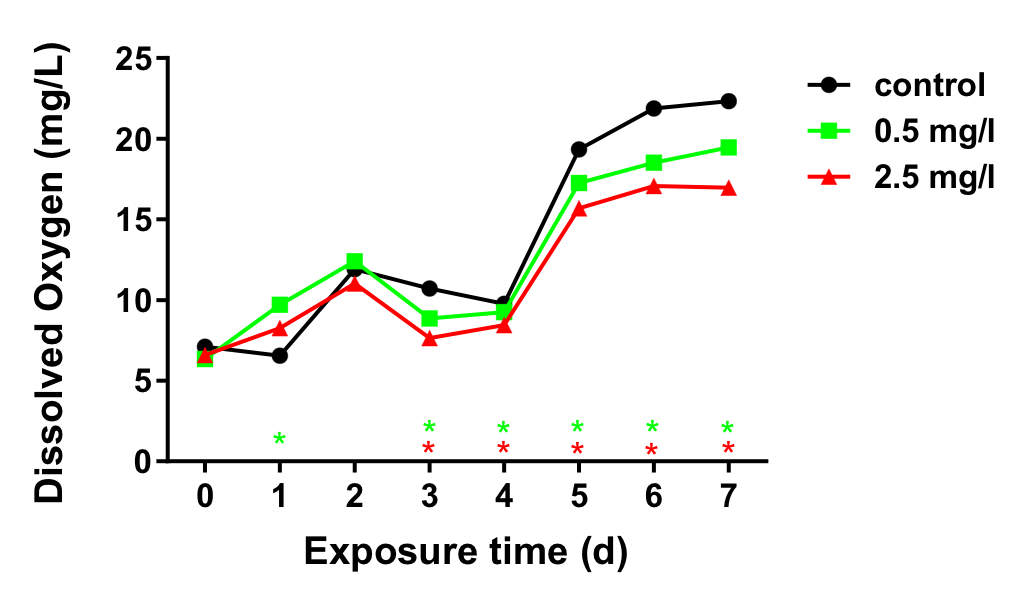


**Figure S4. Dissolved oxygen concentration in microcosms**. Dissolved oxygen concentration in microcosms exposed to 0 (control), 0.5 mg/L or 2.5 mg/L (initial concentration of) azoxystrobin for 7 days. The asterisks represent statistically signiﬁcant diﬀerence at p < 0.05 between control and the AZ-treated microcosms.


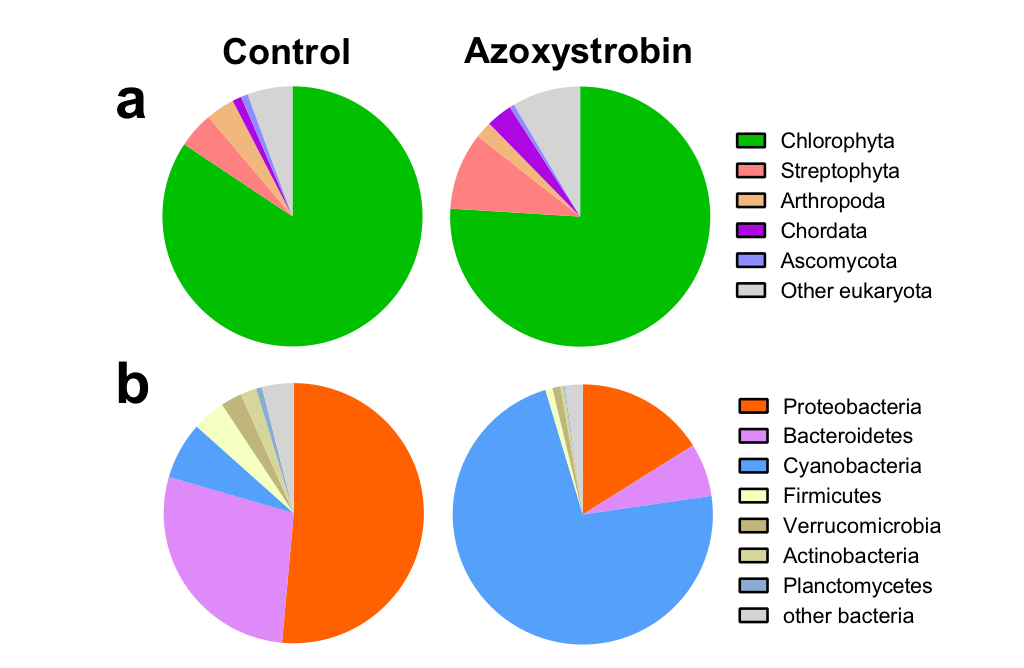


**Figure S5.** **Taxonomic proportions of transcripts in eukaryotic and bacterial community.** Taxonomic proportions of transcripts in eukaryotic (a) and bacterial (b) community in control and AZ-treated microcosms.


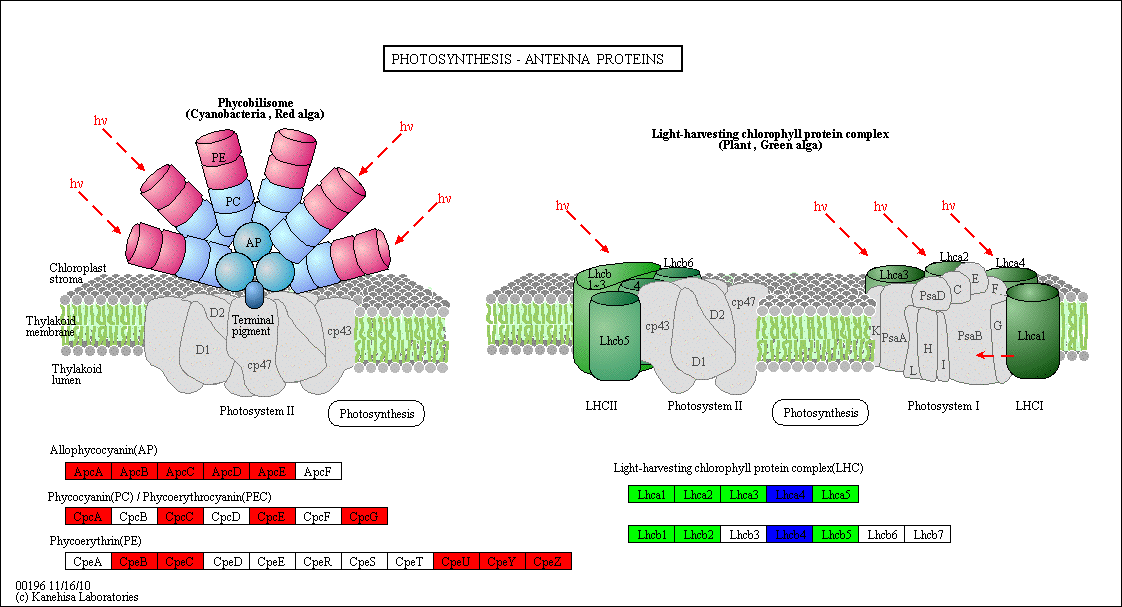


**Figure S6. Gene expression variations in photosynthesis – antenna proteins.** Photosynthesis – antenna protein of the photosystem Ⅱ (pathway ko00196, photosynthesis – antenna proteins) after the 7-day exposure to 2.5 mg/L initial AZ concentrations in the microcosms. Most genes involved in phycobilisome synthesis (Cyanobacteria) were over-expressed (detailed information in dataset 5). The red- or blue-ﬁlled boxes indicate genes encoding this protein upregulated or downregulated after 7 days, respectively. The green-ﬁlled boxes represent that their exist both upregulated and downregulated genes related to the protein after 7 days. The colourless boxes are protein with no signiﬁcant changes in expression after 7 days.

**Figure S7. Bacterial community variation after 7d-culture.** The origin bacterial community (16S rRNA gene sequencing results) and the relative abundance of taxonomically annotated sequences in the bacteria after 7d-culture in the control and AZ treated groups (metatranscriptomic sequencing results).


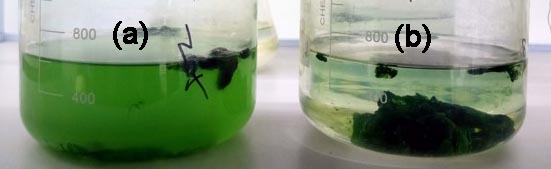

**Figure S8. Long-term impact of AZ contamination.** After 50 days of AZ treatment (initial concentration 2.5mg/L), AZ group (a) still showed the typical characteristics of algal bloom, which appeared green and turbid; while in the control group (b), the solid contents were settling and the microcosm looked clear.

**Figure S9.** **Functional variations between the control and azoxystrobin (AZ) group at KEGG level 2.** Mean relative abundance of functional categories in the control group (Con) or the microcosms exposed to 2.5 mg L^−1^ AZ (initial concentrations) for 7 days (AZ) based on meta-transcriptomic analysis. The asterisks represent statistically signiﬁcant differences at p < 0.05 between the control group and the AZ group.

**Table S1. Chemical composition of the Modified BG11 medium.**

| Chemical | Concentration (mg/L) |
| --- | --- |
| NaNO_3_  MgSO_4_·7H_2_O | 150  75 |
| Citric acid | 6 |
| Ferric ammonium citrate | 6 |
| EDTA-Na_2_ | 1 |
| CaCl_2_·2H_2_O | 36 |
| K_2_HPO_4_ | 4 |
| Na_2_CO_3_ | 20 |
| H_3_BO_4_ | 2.86 |
| MnCl·4H_2_O | 1.81 |
| ZnSO_4_ | 0.222 |
| Na_2_MoO_4_ | 0.39 |
| CuSO_4_·5H_2_O | 0.079 |
| Co(NO_3_)_2_·6H_2_O | 0.049 |

**Table S2.** **Taxonomic proportions of FPKM normalized transcript counts at the phylum level in the control and AZ-treated microcosms.**

|  | Taxon | Control (%) | AZ (%) | Fold change (AZ/Control) |
| --- | --- | --- | --- | --- |
| Algae and photosynthetic  bacteria | Chlorophyta | 63.599 | 35.770 | 0.562 |
|  | Cyanobacteria | 1.708 | 38.344 | 22.447 |
|  | Chloroflexi | 0.106 | 0.045 | 0.426 |
|  | Phaeophyceae | 0.081 | 0.034 | 0.415 |
|  | Chlorobi | 0.042 | 0.329 | 7.742 |
|  | Eustigmatophyceae | 0.112 | 0.053 | 0.475 |
|  | Streptophyta | 3.353 | 4.622 | 1.378 |
|  | Bacillariophyta | 0.032 | 0.203 | 6.321 |
| Zooplankton | Arthropoda | 2.688 | 1.065 | 0.396 |
|  | Nematoda | 0.076 | 0.079 | 1.042 |
|  | Platyhelminthes | 0.051 | 0.031 | 0.611 |
|  | Cnidaria | 0.035 | 0.027 | 0.783 |
|  | Chromerida | 0.035 | 0.015 | 0.430 |
| Fungi | Ascomycota | 0.661 | 0.299 | 0.453 |
|  | Basidiomycota | 0.143 | 0.084 | 0.583 |
|  | Mucoromycota | 0.111 | 0.075 | 0.675 |
|  | Chytridiomycota | 0.069 | 0.037 | 0.532 |
| Bacteria | Proteobacteria | 12.599 | 8.388 | 0.666 |
|  | Bacteroidetes | 6.899 | 3.437 | 0.498 |
|  | Firmicutes | 1.000 | 0.459 | 0.459 |
|  | Verrucomicrobia | 0.647 | 0.517 | 0.799 |
|  | Armatimonadetes | 0.047 | 0.419 | 8.871 |
|  | Planctomycetes | 0.183 | 0.073 | 0.399 |
|  | Candidatus Woesebacteria | 0.102 | 0.026 | 0.256 |
|  | Nitrospirae | 0.028 | 0.011 | 0.377 |
|  | Actinobacteria | 0.487 | 0.206 | 0.424 |
|  | Acidobacteria | 0.037 | 0.021 | 0.560 |
|  | Thermotogae | 0.181 | 0.061 | 0.335 |
|  | Deinococcus-Thermus | 0.164 | 0.117 | 0.711 |
| Archaea | Euryarchaeota | 0.034 | 0.029 | 0.855 |

**Table S3. Variation of pathways related to glycan synthesis and degradation.** Relative abundance (%) of pathways related to glycan synthesis and degradation in bacteria, eukaryote and total biomass in control (Con) and AZ-treated microcosms (AZ) for 7 days.

| KEGG pathways (level 3) | Bacteria | | | Eukaryota | | | Total | | |
| --- | --- | --- | --- | --- | --- | --- | --- | --- | --- |
|  | Con | AZ | FC | Con | AZ | FC | Con | AZ | FC |
| N-Glycan biosynthesis | 0.052 | 0.015 | 0.29 | 0.015 | 0.024 | 1.64 | 0.022 | 0.020 | 0.93 |
| Other glycan degradation | 0.071 | 0.751 | 10.60 | 0.007 | 0.015 | 2.03 | 0.020 | 0.366 | 18.64 |
| Various types of N-glycan biosynthesis | 0.011 | 0.003 | 0.23 | 0.054 | 0.127 | 2.36 | 0.046 | 0.069 | 1.48 |
| Other types of O-glycan biosynthesis | 0.007 | 0.006 | 0.76 | 0.004 | 0.016 | 3.53 | 0.005 | 0.011 | 2.20 |
| Lipopolysaccharide biosynthesis | 0.259 | 0.097 | 0.37 | 0.002 | 0.005 | 2.36 | 0.050 | 0.049 | 0.98 |
| Peptidoglycan biosynthesis | 0.438 | 0.118 | 0.27 | 0.000 | 0.001 | 4.53 | 0.082 | 0.056 | 0.69 |

FC: foldchange(AZ/Con)

**Table S4. Variation of pathways related to vitamin synthesis and consumption.**  Relative abundance (%) of pathways related to vitamin synthesis and consumption in bacteria, eukaryote and total biomass in control (Con) and AZ-treated microcosms (AZ) for 7 days.

| KEGG pathways (level 3) | Bacteria | | | Eukaryota | | | Total | | |
| --- | --- | --- | --- | --- | --- | --- | --- | --- | --- |
|  | Con | AZ | FC | Con | AZ | FC | Con | AZ | FC |
| Thiamine metabolism | 0.282 | 0.385 | 1.365 | 0.392 | 0.701 | 1.789 | 0.370 | 0.551 | 1.490 |
| Riboflavin metabolism | 0.105 | 0.049 | 0.461 | 0.008 | 0.014 | 1.712 | 0.027 | 0.031 | 1.161 |
| Nicotinate and nicotinamide metabolism | 0.452 | 0.742 | 1.641 | 0.551 | 0.629 | 1.142 | 0.532 | 0.683 | 1.283 |
| Pantothenate and CoA biosynthesis | 1.532 | 1.188 | 0.776 | 0.121 | 0.276 | 2.277 | 0.382 | 0.709 | 1.859 |
| Vitamin B6 metabolism | 0.064 | 0.046 | 0.725 | 0.021 | 0.040 | 1.914 | 0.029 | 0.043 | 1.490 |
| Biotin metabolism | 0.065 | 0.025 | 0.386 | 0.059 | 0.067 | 1.141 | 0.060 | 0.047 | 0.789 |
| Lipoic acid metabolism | 0.104 | 0.033 | 0.318 | 0.001 | 0.005 | 3.430 | 0.021 | 0.018 | 0.891 |
| Folate biosynthesis | 0.306 | 0.730 | 2.387 | 0.021 | 0.031 | 1.502 | 0.074 | 0.357 | 4.797 |

FC: foldchange(AZ/Con)
